# Social capital is associated with improved subjective well-being of older adults with chronic non-communicable disease in six low- and middle-income countries

**DOI:** 10.1186/s12992-019-0538-y

**Published:** 2020-01-02

**Authors:** Aaron K. Christian, Olutobi Adekunle Sanuade, Michael Adu Okyere, Kafui Adjaye-Gbewonyo

**Affiliations:** 1 Regional Institute for Population Studies (RIPS), University of Ghana, P.O. Box LG 96, Accra, Ghana; 2 NCDs Support Centre for Africa, Noguchi Memorial Institute for Medical Research, University of Ghana, Accra, Ghana; 3 School of Management China, Institute for Studies in Energy Policy (CISEP), Xiamen University, Xiamen, China; 4 Institute of Advanced Studies, University College London, London, UK

**Keywords:** Chronic non-communicable disease, Social capital, Older adults

## Abstract

**Background:**

Non-communicable diseases (NCDs) are increasingly contributing to the morbidity and mortality burden of low and-middle income countries (LMIC). Social capital, particularly participation has been considered as a possible protective factor in the prevention and management of chronic conditions. It is also largely shown to have a negative effect on the well-being of patients. The current discourse on the well-being of individuals with NCDs is however focused more on a comparison with those with no NCDs without considering the difference between individuals with one chronic condition versus those with multiple chronic conditions (MCC).

**Method and objective:**

We employed a multinomial logit model to examine the effect of social capital, particularly social participation, on the subjective well-being (SWB) of older adults with single chronic condition and MCC in six LMIC.

**Findings:**

Social capital was associated with increased subjective well-being of adults in all the six countries. The positive association between social capital and subjective well-being was higher for those with a single chronic condition than those with multiple chronic conditions in India and South Africa. Conversely, an increase in the likelihood of having higher subjective well-being as social capital increased was greater for those with multiple chronic conditions compared to those with a single chronic condition in Ghana.

**Discussion:**

The findings suggest that improving the social capital of older adults with chronic diseases could potentially improve their subjective well-being. This study, therefore, provides valuable insights into potential social determinants of subjective well-being of older adults with chronic diseases in six different countries undergoing transition. Additional research is needed to determine if these factors do in fact have causal effects on SWB in these populations.

## Introduction

Non-communicable diseases (NCDs) continue to have a heavy toll on the morbidity and mortality rates of low- and middle income countries (LMICs) [1, 2]. NCDs refer to clinical conditions excluding injuries, maternal and perinatal conditions and nutritional deficiencies that do not involve an acute infection or parasite, hence are not transmittable from person to person [3]. They are often characterized by being chronic, leaving residual disability, causing nonreversible pathological alteration and requiring special training of the patient for rehabilitation. They may also require a long period of supervision, observation, or care” [4]. These conditions, however, managed, affect the psychological wellbeing of patients in varying ways.

Although no age-group is immune to NCDs, population ageing has led to a significant rise in the NCD burden compared to other human pathologies [5]. The subjective wellbeing and quality of life of individuals may be intricately linked to their health conditions, more so among older adults, as the incidence of NCDs increases in his group [6]. Age is an important risk factor for several NCDs [7]. The growing recognition of well-being in the health policy scholarship has fueled research to examine the relationship between subjective well-being or quality of life and health outcomes such as NCDs [8]. However, findings from these studies have been inconsistent. For example, whereas greater well-being was positively associated with lower incidence of cancer, Type 2 diabetes and cardiovascular disease [9–11], some other studies found no such relationship [12, 13]. Thus, there is a need for more studies on this issue, as well as examining possible moderating factors of the association between well-being and NCDs, such as social capital.

Social capital can be described as ‘the ability of actors (individuals or groups) to secure benefits by virtue of membership in social networks and other social structures’ [14]. In their seminal work on ‘Social integration, social networks, social support, and health’, Berkman and Glass stipulated that social capital may influence health by serving as a ‘buffering factor’ for stress, influencing particular lifestyle behaviors such as smoking and drinking and provide opportunities to learn new skills and information about disease state [15]. A systematic review of the relationship between social capital and NCDs found a positive association between social capital and the prevention of NCDs [16].

**Fig. 1:**
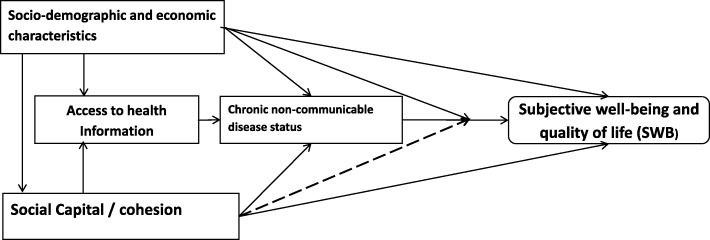
Conceptual framework showing the relationship between social capital, chronic diseases and well-being

Succinctly, the relationship between social capital and the well-being of individuals with NCDs is illustrated and hypothesized in Fig. 1. That sociodemographic and economic status (SES) or variables such as income, employment status, age, education are shown to influence on the well-being of individuals is not contested [17–19]. SES may influence the well-being of individuals with NCDs directly or via alternate pathways shown below -through access to health information [20] or through social capital [21]. Social capital in-turn has been shown to directly influence NCD prevention and general well-being and quality of life [22, 23].

Sparsely reported in the literature, however, is how social capital influences the subjective well-being particularly of older adults with NCDs and whether it moderates (illustrated with broken lines in Fig. 1) the well-being of older adults already with chronic conditions. The current discourse on the well-being of individuals with NCDs is focused more on a comparison with those with no NCDs without considering the difference between individuals with one chronic condition versus those with more than one chronic disease condition. In 2017, the World Economic Forum event together with the United Nations General Assembly stated that, despite the prevalence of Multiple Chronic Conditions (MCCs), there is a scarcity of information on their impact and cost on patients [24]. Arguably MCCs accrue a noticeably higher personal burden on patients with almost a doubling of healthcare cost with any additional chronic condition [25]; thus, they may have a higher toll on the subjective well-being of patients compared to their counterparts with no or single chronic disease.

The objective of this paper is to examine the effect of social capital, particularly social participation, on the subjective well-being of older adults with single NCD and MCC in six LMICs that exhibit some variation in their social interaction. Examination of differences in the predictors of well-being among older adults with NCDs may be useful for understanding the unique needs of older adults in LMICs to promote more effective interventions. In addition, this study has the advantage of using standardized questions on social capital and subjective well-being across representative samples in a range of LMICs. We hope our evidence will help to clarify the relationship between social capital and well-being among individuals with chronic conditions in different countries. To the best of our knowledge, no empirical analysis has examined this question systematically by employing a sample of the world’s population.

## Methods

### Study population and sampling

The data used for this study are from the 2007-to 2010 World Health Organization (WHO) Survey on Global Ageing and Adult health (SAGE). This study collected information from a nationally representative sample in six LMICs-Ghana, China, India, Russia and South Africa- representing different global geographical blocks. Sampled individuals were adults aged 50 years and older and a smaller comparison group of adults between the ages of 18-to 49 years. WHO SAGE used a stratified, multistage cluster sampling design which was based on country-specific censuses. Stratification was mainly based on regions/province and location (urban/rural). Each stratum was then sub-divided into enumeration areas to guide and facilitate coverage. A probability proportional to size (PPS) method was employed to identify the primary sampling units, whilst households were picked randomly within sampling units. Data were collected from 2008 to 2009, 2008 to 2010, 2007 to 2008, 2009 to 2010, 2007 to 2010 and 2007 to 2008 and sample sizes were 5110, 14,813, 11,230, 2756, 4355 and 4223 for Ghana, China, India, Mexico, Russian and South Africa, respectively. Face-to-face interviews were conducted using a structured household roster and an individual questionnaire. Detailed data collection methods have been described by Kowal et al., 2012 and on the SAGE website (www.who.int/healthinfo/system/sage).

### Ethical approval and consent

Ethical clearance and permission for the SAGE study were sought and approved by the Ethics Review Committee, World Health Organization, Geneva, Switzerland and ethics committees and/or institutional review boards in universities and health institutions in participating countries. The SAGE study was approved the Ethics Review Committee, World Health Organization; Ethical Committee and by the following bodies: Human Sciences Research Council, Pretoria, South Africa; and Ethics Committee, National Institute of Public Health (INSP), Cuernavaca, Mexico; Ghana Medical School, Accra, Ghana; Ethics Committee, OPM (School of Preventive and Social Medicine), Russian Academy of Medical Sciences, Moscow, Russia; Ethics Committee, Shanghai Municipal Centre for Disease Control and Prevention, Shanghai, China; Institutional Review Board, International Institute of Population Sciences, Mumbai, India; Research Ethics Committee. Written informed consent was obtained from all participating individuals. The data integrity and respondent records were kept confidential at all times by the SAGE country teams.

### Measures

Table 1 provides a description of the measures (subjective well-being and quality of life, chronic disease status, social capital) and the selected socidemographic and lifestlye characteristics of the respondents that were used in the empirical model.

**Table 1:** Measures used in the analysis of the effects of social capital on the wellbeing of older adults with NCDs

Outcome variable	Health Status	Moderating variable	Covariates
Subjective well-being	Chronic disease	Social capital	Sociodemographic and lifestyle
Respondents were asked to rate their well-being (on a 5 -point scale; from very satisfied (1) to very dissatisfied (5)) with respect to how satisfied they were with: 1. Their daily energy 2. Their money to meet needs 3. Their health 4. Themselves 5. Their ability to perform daily living activities 6. Their personal relationships 7. The conditions of their living place and 8. Their life as a whole	Respondents who had been diagnosed with any one or more of the following diseases: 1. Arthritis 2. Hypertension 3. Stroke 4. Angina 5. Diabetes 6. Chronic lung disease, 7. Asthma 8. Depression	Respondents’ engagement in social activities in the past 12 months. 1. Attended any public meeting in which there was a discussion of local or school affairs 2. Met personally with someone you consider to be a community leader 3. Attended any group, club, society, union or organizational meeting 4. Worked with other people in your neighbourhood to fix or improve something 5. Had friends over to your home? 6. Been in the home of someone who lives in a different neighbourhood than you do or had them in your home 7. Socialized with co-workers outside of work 8. Attended religious services (not including weddings and funerals) 9. Gotten out of the house/your dwelling to attend social meetings, activities, programs or events or to visit friends or relatives The response categories were never (1), once or twice per year (2), once or twice per month (3), once or twice per week (4), and Daily (5).	Respondent characteristics A. Sociodemographic 1. Gender (Male/Female) 2. Age in years 3. Formal education (no formal/primary/ secondary/tertiary education) 4. Married /not married 5. Employment status (currently working/not currently working) 6. Income levels B. Lifestyle characteristics 7. Ever/ Never/Present alcohol consumption 8. Ever/ Never/Present smoked tobacco 9. Daily servings of fruit and vegetable 10. Physical exercise 11. Rural Urban

#### Outcome variable

##### Subjective well-being (SWB)

A summation of scores from the eight questions shown in Table 1 indicates worst possible score (being very dissatisfied with all aspects of one’s well-being) is 8 whilst the best possible score (being very satisfied with all aspect of one’s well-being) is 40. Respondents’ well-being and quality of life scores were divided into tertiles in order to categorize them as ‘Low’, ‘Moderate’ and ‘High’.

#### Main independent variable

##### Chronic disease status

Chronic disease status was based on respondent responses to questions concerning their health problems or health care needs that they may have experienced, and the treatment or medical care received. Chronic diseases classified were arthritis, hypertension, stroke, angina, diabetes, chronic lung disease, asthma and depression.

Respondents were categorised as having no chronic disease (0), one chronic disease [1] and multiple/more than one chronic disease [2]. After determining the prevalence of respondents with NCDs and those without, the sample for the proceeding analyses used only individuals with NCDs i.e. those with a single NCD and those with MCC.

#### Main moderating variable

##### Social capital

Our measure of social capital focusses specifically on social participation. A summation of scores from the nine questions shown in Table 1 indicates worst possible score (having no social capital with respect to engagement in social activities) is 0 whilst the best possible score (being very social active in all 9 social activities) is 36. This variable was entered as a continuous variable in the models.

*Other covariates:* Based on literature and other hypothesised predictors of subjective well-being and /or chronic diseases, the following covariates were controlled for: level of education [26]; current employment status [18]; income levels [17]; marital status [27]; age [28]; fruit and vegetable consumption [29]; rural/urban resident location [30]; alcohol consumption and tobacco consumption [31].

### Estimation technique

A multinomial logit model was used to examine the effect of social cohesion on SWB. This technique allows for the determination of choice probabilities for multiple categories [32]. Unlike ordinal logistic regression, multinomial logistic regression allows for comparisons between multiple outcome categories without requiring the proportional odds assumption of an equivalent strength of association between each consecutive category. The multinomial logistic regression model is defined as follows:

1 $$ P\left(y=j/x\right)=\frac{e^{{x^{\hbox{'}}}_i{\beta}_j}}{\sum_{l=1}^m{e}^{{x^{\hbox{'}}}_i{\beta}_j}}\;j=1,...,J $$

Where *y* denote a random variable taking on the values (1, 2, ..., *J*) for a positive integer *J*, and let *x* denote a set of conditioning variables. In this case *y* and *x* denote the individual’s SWB status and their level of social cohesion, respectively. The question is how, ceteris paribus, changes in the elements of *x* affect the response probabilities *P*(*y* = *j*/*x*), *j* = 1, ..., *J*. Since the probabilities must sum up to unity, *P*(*y* = *j*/*x*) is determined once we know the probabilities for *j* = 2, .., *J*.

For the parameter estimates of the model in equation [1] to be unbiased and consistent, it requires the assumption of independent of irrelevant alternatives (IIA) to hold. More specifically, the IIA assumption requires that the probability of belonging to an SWB category must be independent of the probability of choosing another subjective wellbeing category (that is *P*_*j*_/*P*_*k*_ is independent of the remaining probabilities). The premise of the IIA assumption is the independent and homoscedastic disturbance terms of the basic model in equation [1]. A relative risk ratio (RRR) > 1 indicates that the risk of the outcome falling in the comparison group relative to the risk of the outcome falling in the referent group increases as the variable increases and vice versa. In other words, the comparison outcome is more likely or otherwise.

## Result

### Chronic non-communicable disease status by country of residence

**Fig. 2:**
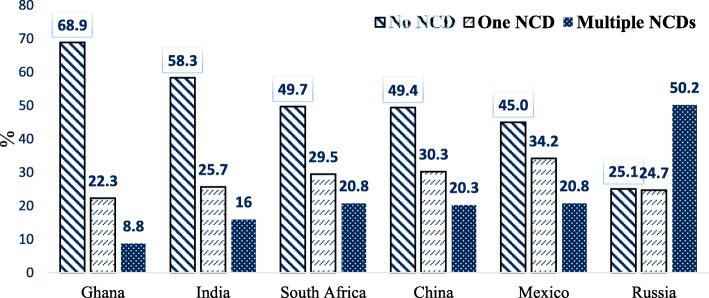
Chronic non-communicable disease status by country of residence

Figures 1 and 2 shows that generally, more than one-fifth of individuals in all the six countries had single or multiple NCDs. Ghana had the lowest proportion of individuals with single or multiple NCDs (22.3 and 8.2%, respectively) and Russia had the highest proportion of individuals with single or multiple NCDs (24.4 and 50.0%, respectively).

### Background characteristics of respondents by chronic disease status

The background characteristics of respondents in all six countries by their NCD status are shown in Table 2. For Ghana, Russia, South Africa and Mexico, more than half of individuals with MCC compared to those with a single NCD were significantly more likely to be females than males. Except for India, the proportion of individuals living with MCC varied by marital status.

**Table 2:** Selected sociodemographic characteristics and lifestyle behaviours in Ghana, China, India, Russia, South Africa and Mexico by NCD status^a^

	Ghana			China			India		
	One NCD	MCC		One NCD	MCC		One NCD	MCC	
	*n* = 929	*n* = 368		*n* = 3751	*n* = 2506		*n* = 1650	*n* = 1032	
Social capital score	14.7 ± 7.2	13.6 ± 7.6	**	5.9 ± 3.5	5.4 ± 3.7	***	8.6 ± 5.0	8.7 ± 5.4	
Sub. Well-Being									
Low	29.7	36.9	**	12.1	20.3	***	22.5	27.6	***
Medium	25.1	23.4		22.9	27.5		24.8	26.8	
High	45.2	39.7		64.9	52.1		52.7	45.5	
Female	55.2	63.9	**	55.6	56.7		51.4	47.7	*
Married	49.9	57.1	**	83.4	79.1	***	73.1	74.7	
Formal Education									**
< Primary	49.5	48.9		24.7	26.3		46.8	43.6	
Primary	21.2	23.4		37.2	35.0		29.6	27.3	
Secondary	24.2	22.8		33.1	32.7		18.3	21.8	
Tertiary	5.1	4.9		5.0	6.1		5.2	7.3	
Currently working	39.0	54.9	***	36.9	21.3	***	35.4	30.0	**
Low income tertile	30.8	27.4	**	39.0	37.2	**	32.5	26.8	***
Age in yrs.	65.5 ± 10.8	68.1 ± 10.2	***	63.4 ± 9.1	67.2 ± 9.1	***	62.5 ± 9.1	63.6 ± 9.0	**
Fruit & Vegetables	4.4 ± 2.6	4.2 ± 2.1		9.4 ± 5.2	9.1 ± 5.0	**	2.8 ± 1.6	3.2 ± 2.	***
Currently takes alcohol	24.4	17.1	***	19.5	13.8	***	7.7	7.0	
Currently Smokes	9.5	6.5		25.6	18.6	***	44.5	44.8	
Moderate exercise ^b^	0.6 ± 1.4	0.9 ± 1.5	**	0.9 ± 2.2	0.9 ± 2.2		0.5 ± 1.7	0.7 ± 1.9	**
	Russia			South Africa			Mexico		
	One NCD	MCC		One NCD	MCC		One NCD	MCC	
	*n* = 890	*n* = 1814		*n* = 922	*n* = 635		*n* = 766	*n* = 577	
Social capital score	6.8 ± 4.1	5.8 ± 4.1		11.5 ± 5.6	11.0 ± 5.7		5.8 ± 4.8	6.1 ± 4.4	
Sub. Well-Being									
Low	13.8	30.1	***	21.2	27.1	**	9.3	12.0	**
Medium	20.1	28.4		24.7	26.8		15.7	21.3	
High	66.1	41.4		54.1	46.1		75.1	66.7	
Female	64.2	70.3	**	58.8	68.4	***	37.1	74.3	***
Married	57.8	50.4	***	51.1	46.0	**	65.4	52.5	***
sFormal Education			**			*			**
< Primary	0.5	1.6		33.5	30.6		18.4	17.5	
Primary	10.6	11.8		42.5	48.5		64.5	65.2	
Secondary	68.6	69.4		19.9	17.0		9.1	7.9	
Tertiary	20.3	17.2		4.1	3.9		8.0	9.4	
Currently working	40.9	20.7	***	27.3	13.9	***	23.9	18.4	**
Low income Tertile	36.4	39.4	**	36.6	26.7	***	43.7	38.8	
Age in yrs.	63.5 ± 10.0	68.1 ± 9.8	***	62.5 ± 9.7	63.8 ± 9.0	**	68.4 ± 9.1	68.7 ± 8.7	
Fruit & Vegetables	3.5 ± 2.5	3.3 ± 2.3	**	3.7 ± 2.2	4.0 ± 2.5	**	3.4 ± 1.9	3.3 ± 1.9	
Currently takes alcohol	35.4	26.5	***	13.1	11.2		13.3	7.8	**
Currently Smokes	20.8	12.8	***	25.6	25.2		17.9	16.5	
Moderate exercise ^b^	0.4 ± 1.5	0.4 ± 1.4		0.2 ± 0.9	0.2 ± 0.9		0.2 ± 0.9	0.2 ± 1.1	

^a^Values present percentages or means ± SD; ^b^Number of times engaging in moderate exercise in a week

Chi-square test, ANOVA was used to examine group differences. ***p < 0.001, **p < 0.05

Significantly more individuals with MCC in Ghana, China, Russia and Mexico were married than unmarried. This was, however, the reverse in South Africa. Excluding Ghana, the proportion of individuals with MCC significantly decreases with increasing education. The mean ages of individuals with Single NCD in all but Mexico compared to their counterparts with MCC were significantly lower.

Fruit and vegetable consumption was highest in China and lowest in India. Individuals with single NCDs in China and Russia consumed significantly more mean number of fruits and vegetables compared to their counterparts with MCC, however, their counterparts in India and South Africa consumed significantly less. With the exception of India and South Africa, respondents from the other LMIC with single NCD’s consumed more alcohol than their counterparts with MCC. On the average individuals in Ghana with MCC exercised more than those with single NCDs.

### Effect of social capital and other explored covariates on the subjective well-being of older adults with NCDs

Tables 3 and 4 presents the results of the regressions, modelling the subjective wellbeing of individuals with chronic diseases against social capital. Results for the coefficient of determination (Psuedo R-Sq.) indicates about 15.0, 9.0, 12.0, 10.0, 9.0 and 4.0% of the variability in SWB is explained by the regressors in Ghana, China, India, Russia, South Africa and Mexico respectively.

**Table 3:** Multinomial logit regression results of social capital and subjective well-being (SWB) among older adults in LIMC’s

Explanatory variables	Ghana		China		India	
	Mod. SWB^b^	High.SWB^c^	Mod. SWB	High. SWB	Mod. SWB	High. SWB
Social capital	1.03**	1.08***	1.09***	1.15***	1.12***	1.14***
	(0.02)	(0.02)	(0.02)	(0.02)	(0.02)	(0.02)
MCC^**a**^ (=1, 0 = Single NCD)	0.68	0.63	0.76*	0.49***	1.04	0.87
	(0.24)	(0.24)	(0.12)	(0.07)	(0.24)	(0.19)
Social capital*MMC	1.01	1.02	0.98	0.97	0.97	0.95**
	(0.02)	(0.02)	(0.03)	(0.02)	(0.02)	(0.02)
Male (=1, 0 = Female)	0.69*	0.97	0.88	0.93	0.67**	0.62***
	(0.15)	(0.20)	(0.10)	(0.10)	(0.10)	(0.09)
Married (=1, 0 = Single)	1.13	1.04	1.35***	1.61***	1.06	1.42***
	(0.23)	(0.21)	(0.15)	(0.17)	(0.15)	(0.19)
Age in years	0.98*	0.97***	1.01***	1.03***	0.99**	0.97***
	(0.01)	(0.01)	(0.01)	(0.01)	(0.01)	(0.01)
Working (=1, 0 = not working)	2.29***	3.38***	1.74***	2.19***	2.09***	2.51***
	(0.41)	(0.60)	(0.20)	(0.24)	(0.32)	(0.36)
Income tertile (Ref = Low)						
Medium	0.98	1.64**	1.40***	2.08***	1.33*	1.43**
	(0.22)	(0.37)	(0.17)	(0.23)	(0.21)	(0.22)
High	1.77***	4.17***	1.96***	4.17***	2.69***	4.51***
	(0.36)	(0.86)	(0.22)	(0.44)	(0.40)	(0.64)
Education (ref = No education)						
Primary	0.93	0.82	1.29**	1.39***	1.11	1.23
	(0.20)	(0.17)	(0.14)	(0.14)	(0.15)	(0.16)
Secondary	1.37	1.28	1.50***	1.67***	1.24	1.79***
	(0.33)	(0.29)	(0.21)	(0.22)	(0.25)	(0.34)
Tertiary	1.43	2.16*	1.01	2.39***	1.27	3.32***
	(0.73)	(0.98)	(0.33)	(0.68)	(0.53)	(1.23)
Currently drinking alcohol (=1, 0 = not drinking alcohol)	1.18	1.05	1.45***	1.56***	1.52	1.70**
	(0.24)	(0.21)	(0.21)	(0.21)	(0.40)	(0.42)
Currently smoking (=1, 0 = not drinking alcohol)	0.80	0.61*	0.89	0.79*	1.10	1.04
	(0.23)	(0.17)	(0.11)	(0.09)	(0.14)	(0.12)
Number of fruit and vegetables consumed	0.91***	0.94**	1.02*	1.02**	1.05	1.14***
	(0.03)	(0.03)	(0.01)	(0.01)	(0.04)	(0.04)
Engaged in moderate intensive exercise (=1, 0 = Not engaged)	0.96	1.32***	1.12***	1.13***	1.02	1.06
	(0.08)	(0.09)	(0.03)	(0.03)	(0.04)	(0.04)
Urban (=1, 0 = rural)	1.09	1.33	1.70***	2.17***	1.21	1.33**
	(0.20)	(0.23)	(0.18)	(0.22)	(0.18)	(0.18)
Constant	1.41	1.20	0.14***	0.05***	0.55	0.93
	(0.97)	(0.82)	(0.06)	(0.02)	(0.29)	(0.46)
Pseudo R-sq.	0.15		0.09		0.12	
LR-ChiSq	412.6***		985.3***		650.1***	
Observations	1254		5893		2633	

^a^Multiple Chronic Condition: ^b^Moderate Subject well-being; ^c^Moderate Subject well-being; Robust standard errors in parentheses *** *p* < 0.01, ** *p* < 0.05, * *p* < 0.1

**Table 4:** Multinomial logit regression results of social capital and subjective well-being (SWB) among older adults in LIMCs

Explanatory variables	Russia		South Africa		Mexico	
	Mod. SWB^b^	High.SWB^c^	Mod. SWB	High. SWB	Mod. SWB	High. SWB
Social capital	1.14***	1.19***	1.08***	1.06***	1.01	1.06*
	(0.05)	(0.04)	(0.02)	(0.02)	(0.04)	(0.03)
MCC^a^ (=1, 0 = Single NCD)	0.95	0.30***	1.45	0.62	1.01	0.81
	(0.26)	(0.08)	(0.48)	(0.19)	(0.35)	(0.24)
Social capital*MMC	0.96	1.02	0.95*	0.99	1.01	0.97
	(0.04)	(0.04)	(0.03)	(0.03)	(0.05)	(0.04)
Male (=1, 0 = Female)	1.01	1.16	1.06	0.89	1.15	0.87
	(0.17)	(0.18)	(0.19)	(0.15)	(0.32)	(0.21)
Married (=1, 0 = Single)	1.09	1.19	1.55**	1.47**	1.23	1.04
	(0.15)	(0.16)	(0.27)	(0.23)	(0.30)	(0.22)
Age in years	0.99	1.00	1.00	1.01	0.99	1.00
	(0.01)	(0.01)	(0.01)	(0.01)	(0.01)	(0.01)
Working (=1, 0 = not working)	0.76	1.25	1.36	1.69***	1.59	2.64***
	(0.15)	(0.22)	(0.29)	(0.32)	(0.56)	(0.82)
Income tertile (Ref = Low)						
Medium	1.21	1.46**	1.03	2.07***	1.23	1.52
	(0.19)	(0.22)	(0.21)	(0.38)	(0.38)	(0.41)
High	1.24	1.84***	1.48**	4.03***	1.28	1.73**
	(0.19)	(0.26)	(0.28)	(0.69)	(0.34)	(0.39)
Education (ref = No education)						
Primary	0.68	2.00	1.01	1.01	1.33	1.15
	(0.31)	(1.35)	(0.17)	(0.16)	(0.37)	(0.27)
Secondary	0.83	2.93	1.34	1.77**	1.60	1.41
	(0.36)	(1.94)	(0.33)	(0.40)	(0.81)	(0.62)
Tertiary	1.05	3.90**	1.06	1.94	1.24	1.46
	(0.49)	(2.66)	(0.58)	(0.92)	(0.67)	(0.65)
Currently drinking alcohol (=1, 0 = not drinking alcohol)	1.16	1.22	0.69	0.94	0.77	1.19
	(0.18)	(0.18)	(0.18)	(0.21)	(0.32)	(0.42)
Currently smoking (=1, 0 = not drinking alcohol)	1.04	0.98	0.78	1.13	0.92	0.67
	(0.22)	(0.19)	(0.15)	(0.19)	(0.27)	(0.17)
Number of fruit and vegetables consumed	1.12***	1.14***	0.98	1.01	0.92	1.08
	(0.03)	(0.03)	(0.03)	(0.03)	(0.06)	(0.06)
Engaged in moderate intensive exercise (=1, 0 = Not engaged)	1.14**	1.12**	1.06	1.13	1.02	1.02
	(0.06)	(0.06)	(0.13)	(0.12)	(0.12)	(0.10)
Urban	1.06	1.49***	1.41**	2.15***	0.77	0.94
	(0.16)	(0.22)	(0.23)	(0.33)	(0.20)	(0.21)
Constant	1.04	0.18*	0.37	0.14***	2.24	3.46
	(0.81)	(0.16)	(0.24)	(0.08)	(2.37)	(3.14)
Pseudo R-sq.	0.10		0.09		0.04	
LR-ChiSq	514.2***		271.9***		83.9***	
Observations	2255		1523		1297	

^a^Multiple Chronic Condition: ^b^Moderate Subject well-being; ^c^Moderate Subject well-being; Robust standard errors in parentheses *** *p* < 0.01, ** *p* < 0.05, * *p* < 0.1

The results showed that the positive effect of an increase in social capital on moderate and high SWB observed among those with single NCD was diminished among those with MCC; this was statistically significant in the India and South Africa samples. However, in the Ghana sample, the opposite interaction effect was observed; the positive association between high social capital and SWB observed among those with single NCD was greater or amplified among those with MCC, although this was not statistically significant.

Males in Ghana and India significantly had lower odds of having moderate SWB compared to low SWB when compared with their female counterparts. Marital status was significantly associated with SWB in China, India and South Africa; being married was associated with having moderate and high SWB relative to low SWB. With respect to age, the likelihood of having high compared to low SWB decreased with age for respondents with MCC in Ghana and India; each additional year of age was associated with 0.98 (se = 0.01) times and 0.99 (se = 0.01) times the likelihood of high compared to low SWB in Ghana and India, respectively. Contrarily, respondents in China were significantly more likely to have a higher level of SWB as they advance in age.

Generally, individuals who were employed or working were significantly more likely to experience high SWB across all study countries except Russia. Income and education also tended to be positively associated with SWB. Except for Ghana and Mexico. Further, individuals living in urban areas significantly had a higher likelihood of being in the higher SWB category than in the lower SWB category.

Current alcohol consumption generally had a positive effect on SWB although this association was statistically significant only in China and India. Individuals from Ghana and China who were smoking were 0.61 and 0.79 times as likely to be in the high SWB category respectively than the low SWB category. While the number of fruits and vegetables consumed had a positive association with SWB in China, India and Russia, the effect is negative in Ghana; an increase in the number of fruit and vegetable consumptions in Ghana decreases the likelihood of having moderate and high SWB by 0.91 and 0.94 respectively. Moderate exercise had a positive association with the SWB of the respondents, all else equal. Compared with their counterparts who were not partaking in any forms of exercise, individuals from Ghana, China and Russia engaging in at least moderate exercise were 1.32, 1.13 and 1.12 times as likely to be in the high SWB category respectively than the low SWB.

## Discussion

This study examined the possible additive and interaction effects of social capital and chronic disease status on the SWB of adults in six LMICs (i.e. Ghana, South Africa, China, India, Russia, and Mexico). Our study showed that social capital was associated with increased SWB of adults in all the six countries regardless of their chronic disease status. Particularly, the positive association between social capital and SWB was higher for those with a single chronic condition than those with multiple chronic conditions in India and South Africa. On the other hand, the increase in the likelihood of having high SWB as social capital increased was greater for those with multiple chronic conditions compared to those with a single chronic condition in Ghana.

Regardless of the heterogeneity of the measures used to represent social capital, findings of this study are to some extent similar to other studies. Farajzadegan et al. (2012) suggest that ‘trust and solidarity’ and ‘empowerment and political action’, gained through the various dimensions of social capital influence how individuals manage their chronic disease condition. Research shows that social capital is positively associated with the SWB of the elderly [33, 34]. Also, social capital is important for self-management of chronic diseases even when other socioeconomics characteristics have been controlled for [35]. These explanations are coherent with the assertion by Cockerham et al. (2017) that the objective component of social capital actually promotes the ‘looking out for each other’ and the provision of help when an individual is sick, thus improving well-being. Social capital, in this case social participation, may have a positive effect on the SWB of individuals with NCDs by contributing to NCD self-management [36]. Further, social capital may stimulate social control over unhealthy behaviours of individuals with NCDs and also facilitate social support which has been shown to influence health through emotional support, instrumental support, or informational support [37, 38]. Additionally, social capital enhances communication and the uptake of new information which can positively impact health [39].

Due to the probable increased health care burden of individuals with multiple chronic diseases compared to their counterparts with single chronic conditions, they may have a higher appreciation of their involvement in social groups, as observed in Ghana.

### Other correlates of well-being and quality of life

This study showed that being married was associated with high SWB. Previous studies indicate that the relationship between marriage and well-being is mixed. There are existing studies that have identified that being married could lower subsequent levels of functional limitations among individuals with NCDs and that marital status may be important for physical health [40, 41]. This is believed to be a result of the social support provided by couples to each other and the presence of another person to help and provide for the other’s needs [42]. This scenario, however, is not a given since being married itself may not always mean receiving support; the quality of the relationship also matters. This probably explains the absence of a positive effect of being married among some of the samples in our study.

Compared to individuals not working, those who were working across all the countries were more likely to have higher SWB except for Russia. There is a general consensus that unemployment does have a negative impact on SWB [43, 44], potentially through its impacts on socioeconomic status and resources. Furthermore, Creed and Evans (2002) also found that being unemployed was associated with declines in well-being and this was not a result of reverse causality [45]. Higher education was associated with high SWB and this is consistent with other studies among older adults. This can be attributed to the fact that higher education usually equips older people with the requisite skills and resources, thus enabling them to better adjust their changing socioeconomic status [46].

Our results showed that the SWB of males in the Ghana and India samples was lower than that of their female counterparts. This finding concurs with theories such as the “role of preferences” or “role of expectation”. “Role of preferences” suggests that the higher SWB of women is because women have broad preferences in activities. On the other hand, ‘role of expectation’ suggests that women have higher SWB because they have lower expectations [47, 48]. The fact that there was no difference in SWB for males and females in China, Russia, South Africa and Mexico reveals the need to consider contextual issues affecting SWB across different geographical and cultural space.

### Limitations

This study is not without limitation. Firstly, sample sizes for some comparisons in some of the countries may have been limited or not provided enough power to detect some effects. There are also potential limitations in variable definitions. For example, the chronic disease status was based on self-reports of prior diagnosis which may have the potential for bias if respondents do not give accurate or truthful responses. In addition, there may be differential access to healthcare services based on socio-economic status and other factors. It is therefore likely that there is an under-diagnosis of chronic disease in some social strata. While our models included controls for various social and demographic factors (education, employment), there may be other factors that were unaccounted for that could bias the results. Our analysis also focused on just one aspect of social capital, which was social participation at the individual level. It is possible that other forms of social capital, such as cognitive social capital or contextual measures of social capital, may have different relationships to SWB among individuals with chronic disease. Finally, because the data are from a cross-sectional observational study, we cannot determine causality from the results observed here. It is possible for example, that greater SWB enhances social participation in this sample (reverse causation).

## Conclusion

Using nationally representative data from six LMICs, this paper provides a unique opportunity to investigate the relationship between chronic disease status and the SWB of older adults and how their social capital modulates this relationship. Higher social capital scores, namely more social interactions and social participation are associated with better SWB and this is higher among those with a single chronic condition in all the six countries except Ghana. While we cannot conclude from this result that improving social capital among older adults with NCDs will lead to improvements in SWB, the positive association we observed suggests that social capital is a potential avenue for further research into ways to improve SWB among older adults with chronic conditions. This study, therefore, provides valuable insights into potential social determinants of the well-being of older adults with chronic diseases in six different countries.

## Data Availability

The WHO sage data is already in the Public Domain and can be accessed.
